# Tuberculosis Predicts Increased Mortality in Patients With SARS-CoV-2 Infection

**DOI:** 10.7759/cureus.117825

**Published:** 2026-10-12

**Authors:** Kiran Bala, Pragati Grover, Raunak Bir, Jaya Biswas, Tamanna Bordoloi, Jamshed Nayer, Anant Mohan, Naveet Wig, Randeep Guleria, Urvashi Singh

**Affiliations:** 1 Microbiology, All India Institute of Medical Sciences, New Delhi, IND; 2 Emergency Medicine, All India Institute of Medical Sciences, New Delhi, IND; 3 Pulmonary, Critical Care and Sleep Medicine, All India Institute of Medical Sciences, New Delhi, IND; 4 Internal Medicine, All India Institute of Medical Sciences, New Delhi, IND; 5 Pulmonary Medicine and Sleep Disorders, All India Institute of Medical Sciences, New Delhi, IND

**Keywords:** coinfection, covid-19, genexpert, survival, tuberculosis

## Abstract

Background: Tuberculosis (TB) remains a major public health issue in high-burden countries like India. During the COVID-19 pandemic, disruption of TB services together with overlapping clinical manifestations complicated timely diagnosis of TB among patients infected with COVID-19.

Methods: The prospective observational study was conducted at a North Indian tertiary care centre between April 2020 and June 2021. Laboratory-confirmed COVID-19 patients suspected of TB were evaluated using Ziehl-Neelsen smear microscopy, GeneXpert MTB/RIF (Cepheid, Sunnyvale, CA, USA), in-house PCR, and MGIT-960 culture (Becton Dickinson, Franklin Lakes, NJ, USA). Clinical characteristics, microbiological findings, treatment and outcomes were recorded. Kaplan-Meier survival curves were compared using log-rank test.

Results: Among 155 hospitalized COVID-19 patients evaluated, TB was microbiologically confirmed in 24 (15.5%). Pulmonary TB constituted 66.7% of cases, while 33.3% had extrapulmonary disease. GeneXpert confirmed TB in 17 patients, PCR in six, and MGIT culture in one patient. Mortality was significantly higher in patients coinfected with TB-COVID than in patients with COVID-19 disease alone (37.5% vs 18.3%). Kaplan-Meier analysis showed significantly poorer survival among coinfected patients (log-rank χ² = 6.678, *p* = 0.0097). Severe COVID-19 was independently associated with markedly reduced survival (log-rank χ² = 224.82, *p* < 0.001).

Conclusions: COVID-19-hospitalized patients with TB coinfection succumbed more often. To improve early identification and outcomes in high-burden settings, COVID-19 patients with overlapping clinical and radiological symptoms should undergo TB screening.

## Introduction

Coronavirus disease 2019 (COVID-19), caused by SARS-CoV-2, is a cause of global morbidity and mortality despite breakthroughs in vaccines, diagnosis, and clinical care. India experienced one of the highest cumulative burdens of COVID-19 worldwide, underscoring the importance of identifying factors associated with adverse outcomes among hospitalized patients [1,2].

Tuberculosis (TB) continues to remain a major health problem globally, with 10.7 million new cases and 1.23 million deaths estimated in 2024. India has one-fourth of the global TB burden, and TB remains a major cause of sickness and death despite breakthroughs in diagnosis and treatment [3].

TB-COVID coinfection is of particular concern in high-burden settings because both diseases share overlapping clinical and radiological features, often leading to delayed diagnosis and treatment [4,5]. Consequently, distinguishing one disease from the other can be challenging, often leading to delay in diagnosis and appropriate initiation of therapy [4,6]. Moreover, extrapulmonary TB may present with nonspecific manifestations that can easily be overlooked in patients admitted with acute COVID-19 infection [7,8]. In addition, COVID-19 disease and its treatment may increase susceptibility to progression or reactivation of TB, while pre-existing TB may predispose patients to severe COVID-19 and poorer outcomes [4,5,9-11].

TB and SARS-CoV-2 interact in a likely bidirectional way. Active TB may predispose patients to severe COVID-19 through underlying lung damage and impaired cellular immunity, whereas COVID-19 infection and the use of corticosteroids or other immunomodulatory therapies may facilitate progression or reactivation of TB, particularly in endemic settings [4,5,9,10].

In addition, the COVID-19 pandemic disrupted TB control programmes worldwide, resulting in delayed diagnosis, reduced case notifications, and interruption of treatment services, thereby adversely affecting TB control in high-burden countries [3,11,12].

Accumulating evidence indicates that active TB has an association with severe COVID-19 and increased mortality. However, many published studies relied on retrospective datasets or clinically diagnosed TB, with limited microbiological confirmation and few reporting time-to-event survival analyses, particularly from high TB-burden countries [13-15]. Microbiological confirmation of TB is especially important in COVID-19 patients because overlapping clinical features may delay diagnosis and treatment [4,6]. Despite advances in rapid molecular diagnostics, data describing microbiologically confirmed TB-COVID coinfection from India remain limited [6,7].

The primary objective of this study was to determine the proportion of microbiologically confirmed TB among adult hospitalized COVID-19 patients with clinical suspicion of TB. The secondary objectives were to describe the clinical characteristics and site of disease in coinfected patients, to report the yield of GeneXpert MTB/RIF (Cepheid, Sunnyvale, CA, USA), MPT64 PCR, and MGIT 960 culture (Becton Dickinson, Franklin Lakes, NJ, USA), and to compare in-hospital mortality, survival, and inflammatory biomarkers between patients with TB-COVID coinfection and those with COVID-19 alone.

## Materials and methods

Study design and setting

This was a prospective observational study, conducted in the Department of Microbiology, Pulmonary Medicine and Sleep Disorders, Medicine, and Emergency Medicine at a tertiary care center in India, between April 2020 and June 2021. 

Study population

Adult patients aged ≥18 years who had laboratory-confirmed SARS-CoV-2 infection and were clinically suspected to have TB were consecutively enrolled. Clinical TB suspicion was defined by one or more of the following: fever, cough over two weeks, weight loss, night sweats, hemoptysis, unexplained lymphadenopathy, pleural effusion, meningitis, abdominal or spinal TB, radiological findings suggestive of active TB (cavitary lesions, upper lobe infiltrates, miliary nodules, necrotic mediastinal lymphadenopathy, tree-in-bud appearance, or pleural effusion), previous history of TB, history of close interaction with a TB patient, or a high index of clinical suspicion by the treating physician. Patients were excluded if they did not have enough clinical information or if their specimens were not adequate for microbiological investigation.

Clinical data collection

Demographic characteristics, presenting symptoms, comorbidities, previous history of TB, family history of TB, laboratory findings, radiological investigations, microbiological results, treatment received, and clinical outcomes were recorded using a standardized case record form. Patients were categorized as symptomatic or asymptomatic for COVID-19 by the existence or nonexistence of clinical symptoms attributable to SARS-CoV-2 infection at admission. Chest radiography and computed tomography of the chest were performed as part of the diagnostic workup, guided by clinical suspicion and the treating physician’s discretion.

Specimen collection

Appropriate pulmonary and extrapulmonary clinical specimens were collected depending on the suspected site of disease. Pulmonary specimens included sputum, bronchoalveolar lavage, endotracheal aspirate, and gastric aspirate where indicated. Extrapulmonary specimens included cerebrospinal fluid, pleural fluid, ascitic fluid, pus, lymph node aspirates, tissue biopsies, and other body fluids submitted for routine diagnostic evaluation.

Microbiological diagnosis of tuberculosis

All specimens were processed according to standard laboratory protocols. Decontamination of pulmonary and non-sterile extrapulmonary specimens was done using the N-acetyl-L-cysteine-sodium hydroxide (NALC-NaOH) method prior to microscopy and culture [16]. Specimens were tested by GeneXpert MTB/RIF, MPT64 PCR, MGIT 960 culture, and/or Ziehl-Neelsen microscopy as requested by the treating clinician and as permitted by specimen volume; therefore, not every specimen underwent every test, and the number of specimens tested by each method is shown in Table 1. Results were combined at the patient level, and each patient was counted once.

**Table 1:** Baseline demographic, clinical, microbiological characteristics and outcomes of hospitalized COVID-19 patients with clinical suspicion of tuberculosis (n = 155) Data are presented as number (%) unless otherwise indicated. Abbreviations for units used in the first column: n = absolute count; n (%) = count with percentage of total cohort (n = 155); n/N (%) = count as a proportion of the relevant subgroup tested or confirmed, with percentage; years = median age. Tuberculosis (TB) was considered microbiologically confirmed when Mycobacterium tuberculosis complex was detected by GeneXpert MTB/RIF, MPT64 polymerase chain reaction (PCR), or MGIT 960 liquid culture. Pulmonary and extrapulmonary TB classifications were based on the predominant site of disease. ATT included both microbiologically confirmed cases and clinically diagnosed patients who received empirical therapy based on the treating physician's assessment. Abbreviations: RT-PCR, reverse transcription polymerase chain reaction; CB-NAAT, cartridge-based nucleic acid amplification test; TB, tuberculosis; MGIT, Mycobacteria Growth Indicator Tube; PCR, polymerase chain reaction; ATT, anti-tubercular treatment.

Characteristic	Value
A. Demographic and Clinical Characteristics	
COVID-19 patients clinically suspected of TB, n	155
Male sex, n (%)	100 (64.5)
Female sex, n (%)	55 (35.5)
Median age (years)	44.5
Method of SARS-CoV-2 diagnosis	
• Real-time RT-PCR, n (%)	55 (35.5)
• CB-NAAT, n (%)	100 (64.5)
Symptomatic COVID-19, n (%)	135 (87.1)
Asymptomatic COVID-19, n (%)	20 (12.9)
TB diagnosed simultaneously or within 7 days of COVID-19 diagnosis, n (%)	16 (66.7)
TB diagnosed >7 days after COVID-19 diagnosis, n (%)	8 (33.3)
Microbiologically confirmed TB, n (%)	24 (15.5)
Pulmonary TB, n (%)	16 (66.7)
Extrapulmonary TB, n (%)	8 (33.3)
• Central nervous system TB, n	3
• Lymph node TB, n	1
• Spinal TB, n	2
• Pleural TB, n	1
• Cold abscess, n	1
Previous history of TB, n (%)	4 (2.6)
Family history of TB, n (%)	11 (7.1)
Underlying comorbidities	
Diabetes mellitus, n	3
Hypertension, n	3
Bronchogenic malignancy, n	1
Cryptococcal diarrhoea, n	1
Human immunodeficiency virus infection, n	1
Renal transplantation, n	2
Chronic liver disease, n	2
Chronic kidney disease, n	2
Hepatitis C virus infection, n	1
Acute lymphoblastic leukemia, n	1
Hematological malignancy, n	1
Mucormycosis, n	1
Seizure disorder, n	1
B. Microbiological Diagnosis of Tuberculosis	
Pulmonary specimens tested, n	88
Extrapulmonary specimens tested, n	67
Ziehl–Neelsen smear positive	
• Pulmonary specimens, n/N	0/1
• Extrapulmonary specimens, n/N	0/3
GeneXpert MTB/RIF positive, n/N (%)	17/119 (14.3)
• Rifampicin resistant, n/N (%)	1/17 (5.9)
• Rifampicin susceptible, n/N (%)	16/17 (94.1)
• Pulmonary specimens, n/N (%)	12/70 (17.1)
• Extrapulmonary specimens, n/N (%)	5/49 (10.2)
MPT64 PCR positive, n/N (%)	6/24 (25.0)
• Pulmonary specimens, n/N (%)	3/12 (25.0)
• Extrapulmonary specimens, n/N (%)	3/12 (25.0)
MGIT 960 culture positive, n/N (%)	1/8 (12.5)
• Pulmonary specimens, n/N (%)	1/5 (20.0)
• Extrapulmonary specimens, n/N	0/3
Pulmonary specimens positive for M. tuberculosis, n/N (%)	16/88 (18.2)
Extrapulmonary specimens positive for M. tuberculosis, n/N (%)	8/67 (11.9)
C. Treatment and Clinical Outcomes	
Patients receiving anti-tubercular treatment, n/N (%)	31/155 (20.0)
• Microbiologically confirmed TB, n/N (%)	18/24 (75.0)
• Empirical treatment for clinically diagnosed TB, n/N (%)	13/131 (9.9)
Overall mortality	
• Died, n/N (%)	33/155 (21.3)
• Survived, n/N (%)	122/155 (78.7)
Mortality among COVID-19 alone, n/N (%)	24/131 (18.3)
Survival among COVID-19 alone, n/N (%)	107/131 (81.7)
Mortality among TB–COVID coinfection, n/N (%)	9/24 (37.5)
Survival among TB–COVID coinfection, n/N (%)	15/24 (62.5)

Ziehl-Neelsen smear microscopy

Samples after decontamination were stained using the Ziehl-Neelsen technique and microscopy was performed and graded according to National Tuberculosis Elimination Programme (NTEP) guidelines [17]. 

GeneXpert MTB/RIF assay

The GeneXpert MTB/RIF assay was performed following the manufacturer's protocols for the prompt detection of Mycobacterium tuberculosis complex and rifampicin resistance [18].

Mycobacterial culture

Liquid culture was performed using the automated liquid MGIT 960 culture system as per the manufacturer's instructions. Positive cultures were tested for differentiation into Mycobacterium tuberculosis complex and non-tuberculous mycobacteria (MGIT TBc Identification test) [17].

MPT64 polymerase chain reaction

MPT64 PCR was performed on 24 specimens (12 pulmonary and 12 extrapulmonary; Table 1) using an in-house conventional PCR targeting the mpt64 gene, which is specific for Mycobacterium tuberculosis complex. DNA extraction, amplification, and detection of amplified products by agarose gel electrophoresis were carried out according to the protocol previously established and published from our department [19]. Positive and negative controls were included in each run.

Case definitions

Definition of Tuberculosis

TB was considered microbiologically confirmed when M. tuberculosis complex was detected by at least one microbiological method, including GeneXpert MTB/RIF, MPT64 PCR, or MGIT 960 liquid culture. Patients without microbiological confirmation but with compatible clinical, radiological, or histopathological findings who were initiated on empirical anti-tubercular therapy (ATT) by the treating physician were classified as clinically diagnosed TB.

Definition of COVID-19

COVID-19 was confirmed by reverse transcription polymerase chain reaction (RT-PCR)/cartridge-based nucleic acid amplification (CBNAAT) confirmed cases.

Comparison Group

Patients in whom M. tuberculosis complex was not detected in any specimen by any method were classified as having COVID-19 alone. This group included 13 patients with clinically diagnosed TB who received empirical ATT (Table 1).

Definition of Severe COVID-19

Severe COVID-19 was defined according to the clinical management protocol of the Ministry of Health and Family Welfare, Government of India, as a respiratory rate >30/min or oxygen saturation (SpO2) <90% on room air.

Patient management and follow-up

Patients diagnosed with TB received anti-tubercular treatment according to NTEP guidelines and treating physician discretion. Patients without microbiological confirmation but with strong clinical and radiological suspicion received empirical ATT where considered appropriate.

Clinical outcomes, including survival, mortality, and response to treatment, were recorded until discharge or death.

Statistical analysis

Microsoft Excel (Redmond, WA, USA) and IBM SPSS Statistics 26.0 (IBM Corp., Armonk, NY, USA) were used to analyse data. Continuous variables were expressed as mean ± SD or median (interquartile range, IQR), and categorical variables as frequencies and percentages, based on data distribution. Categorical variables were compared using the Pearson chi-square test, or Fisher’s exact test when any expected cell count was less than five; for the comparison of mortality between groups, the odds ratio (OR) with its 95% confidence interval (CI) was also calculated, and, because of the small number of coinfected patients, Fisher’s exact test p-value was also reported. For continuous data, normality was assessed using the Shapiro-Wilk test; the Student’s t-test was used for normally distributed variables and the Mann-Whitney U test for non-normally distributed variables. All laboratory parameters in Table 2 were non-normally distributed and were therefore summarised as median (IQR) and compared using the Mann-Whitney U test, with results reported as U (Z). Laboratory comparisons were performed at the patient level, using the first available value for each patient; parameters available for fewer than three coinfected patients were summarised descriptively without statistical testing. Survival was analysed using the Kaplan-Meier method, and survival curves were compared using the log-rank test. Survival time was calculated from the date of laboratory confirmation of SARS-CoV-2 infection to death (event) or hospital discharge; patients who were discharged alive were censored at the date of discharge. Patients were assigned to the TB-COVID group if TB was microbiologically confirmed at any time during the index hospitalisation. Because TB was diagnosed after the start of follow-up in some patients, this fixed-group approach is susceptible to immortal-time bias; since coinfected patients necessarily survived until TB was diagnosed, any such bias would favour survival in the TB-COVID group and would therefore tend to underestimate, rather than exaggerate, the survival difference between groups. Two-tailed p values under 0.05 were considered statistically significant.

**Table 2:** Comparison of inflammatory biomarkers and laboratory parameters between patients with COVID-19 alone and TB-COVID coinfection Data are presented as median (interquartile range) with the number of patients (n) for whom each result was available; each patient contributed one value (the first available result). Serum biomarkers were compared using the two-tailed Mann-Whitney U test (U = Mann–Whitney U statistic; Z = standardised normal approximation of U). No statistically significant difference was detected between the groups for any serum biomarker (all p > 0.05); given the small number of coinfected patients, these comparisons had limited statistical power. Cerebrospinal fluid (CSF) parameters were available for only one or two patients with TB-COVID coinfection; individual values are therefore shown and no statistical test was performed (-). IL-6, interleukin-6; IQR, interquartile range.

Parameter	COVID-19 alone, median (IQR); n	TB-COVID, median (IQR); n	Mann-Whitney U (Z)	p value
IL-6 (pg/mL)	26.30 (8.16-69.61); n = 83	56.63 (24.04-121.14); n = 18	960.0 (1.890)	0.059
Ferritin (ng/mL)	455.27 (193.10-805.38); n = 82	345.00 (175.02-640.90); n = 18	618.0 (−1.077)	0.284
C-reactive protein (mg/dL)	3.46 (1.21-10.60); n = 37	3.98 (1.87-15.20); n = 7	149.0 (0.626)	0.542
Procalcitonin (ng/mL)	0.20 (0.07-1.01); n = 74	0.50 (0.12-5.39); n = 14	628.0 (1.255)	0.211
CSF cell count (cells/µL)	20.00 (4.75-165.00); n = 6	5, 35; n = 2	-	-
CSF sugar (mg/dL)	57.50 (20.25-78.25); n = 12	85, 87; n = 2	-	-
CSF lymphocytes (%)	55.00 (22.50-91.25); n = 6	40; n = 1	-	-
CSF polymorphs (%)	45.00 (8.75-77.50); n = 6	60; n = 1	-	-
CSF protein (mg/dL)	89.50 (42.50–119.50); n = 10	29; n = 1	-	-

## Results

Patient characteristics and clinical profile of tuberculosis

During the study period, 155 adult patients with laboratory-confirmed COVID-19 and clinical suspicion of TB were enrolled. Microbiological confirmation of TB was achieved in 24/155 individuals (15.5%). Twenty of the 24 patients with TB had one or more underlying comorbidities. Table 1 demonstrates the demographic characteristics, comorbidities, and clinical profile of the patients. TB was diagnosed in the majority of patients (66.7%) within seven days of laboratory confirmation of COVID-19 disease, while the remaining patients were diagnosed between 10 and 41 days after COVID-19 diagnosis (Figure 1). 

**Figure 1:**
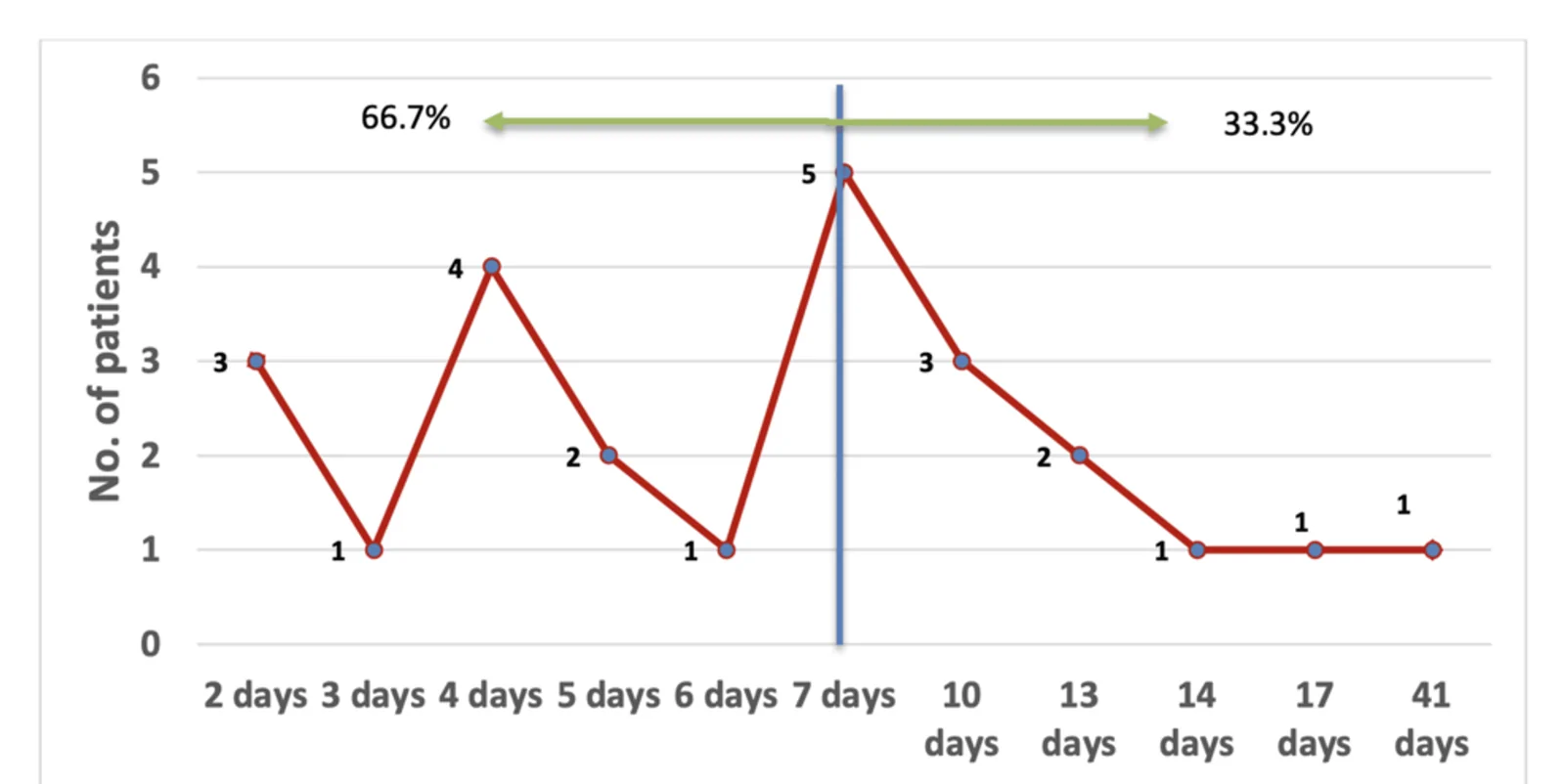
Time interval of diagnosis of tuberculosis after SARS-CoV-2 infection Line graph depicting the time interval between diagnosis of SARS-CoV-2 infection and diagnosis of tuberculosis (TB). Among patients diagnosed with TB, 66.7% were diagnosed within seven days of SARS-CoV-2 diagnosis, while 33.3% were diagnosed after seven days.

Microbiological diagnosis

GeneXpert was the most frequently positive diagnostic test, followed by MPT64 PCR and MGIT culture. Ziehl-Neelsen smear microscopy was performed on only four specimens, all of which were negative. Pulmonary specimens demonstrated a higher positivity rate than extrapulmonary specimens (18.2% vs. 11.9%) (Table 1). 

Treatment and clinical outcome

ATT was initiated in 31 patients, including all microbiologically confirmed cases and those with strong clinical suspicion of TB. Overall in-hospital mortality was 21.3% (33/155). Mortality was higher among patients with TB-COVID coinfection than among those with COVID-19 alone (9/24, 37.5% vs. 24/131, 18.3%; Pearson χ² = 4.45, p = 0.035; Fisher’s exact test, p = 0.054; OR 2.68, 95% CI 1.05-6.83). Six of the nine deaths in the coinfected group occurred within one day of TB diagnosis, before effective ATT could be initiated (Table 1; Figure 2).

**Figure 2:**
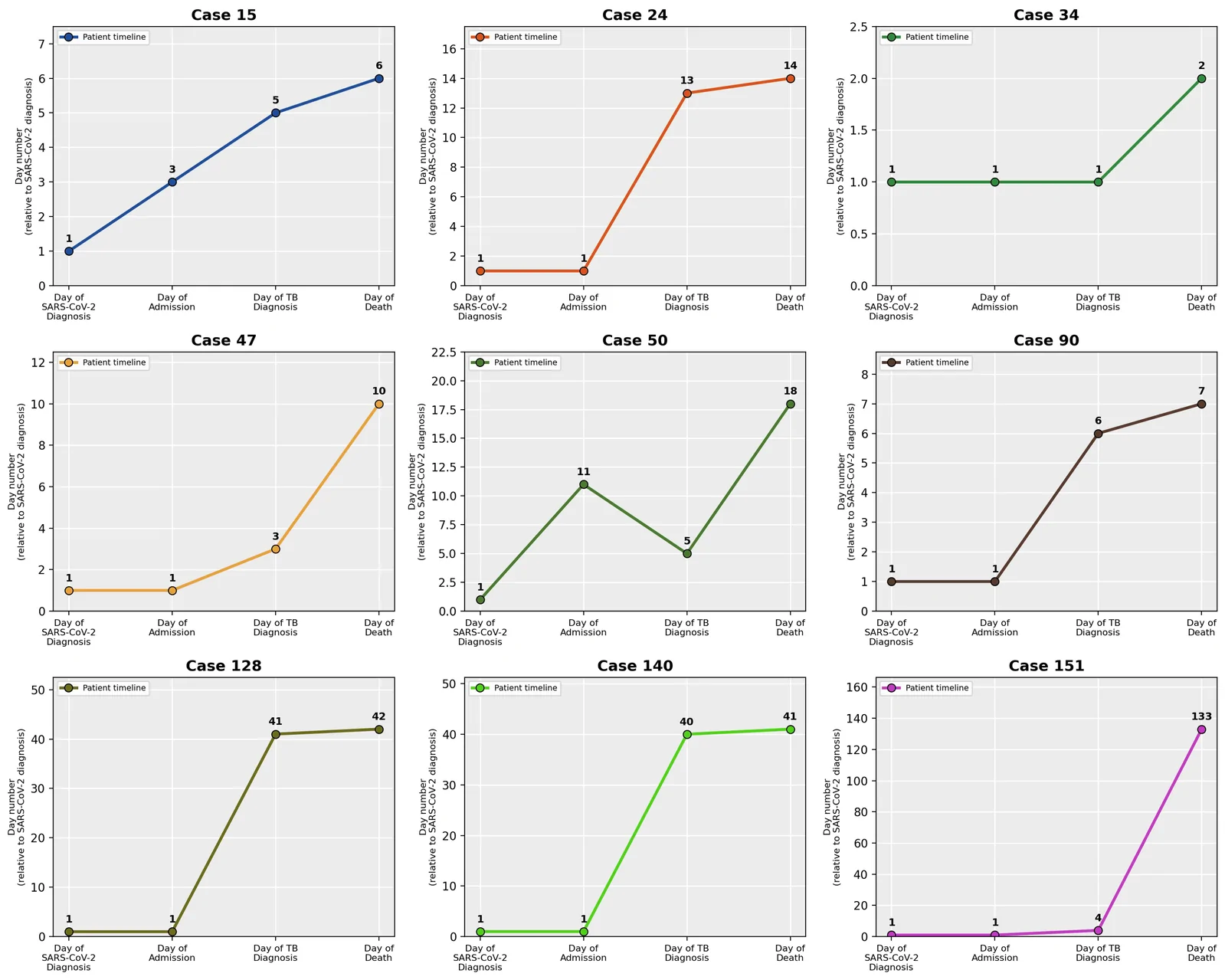
Timeline of tuberculosis diagnosis and death in coinfected patients who died Timeline of key events for patients who died with SARS-CoV-2 and Mycobacterium tuberculosis coinfection. M. tuberculosis was diagnosed within seven days of SARS-CoV-2 diagnosis in cases 15, 34, 47, 50, 90, and 151. Tuberculosis was diagnosed at 13 days in case 24 and at 41 and 40 days in cases 128 and 140, respectively. Six patients died within one day of tuberculosis diagnosis. Cases 47 and 50 died seven and 13 days after tuberculosis diagnosis, respectively, while case 151 died four months after diagnosis. Cases 34 and 90 were diagnosed with meningeal tuberculosis; all other cases were diagnosed with pulmonary tuberculosis.

Survival analysis

TB-COVID coinfection patients had considerably lower survival rates than COVID-19 alone patients, according to Kaplan-Meier analysis (log-rank χ² = 6.678, p = 0.0097) (Figure 3). Similarly, patients with severe COVID-19 had significantly poorer survival than those with non-severe disease (log-rank χ² = 224.82, p < 0.001) (Figure 4).

**Figure 3:**
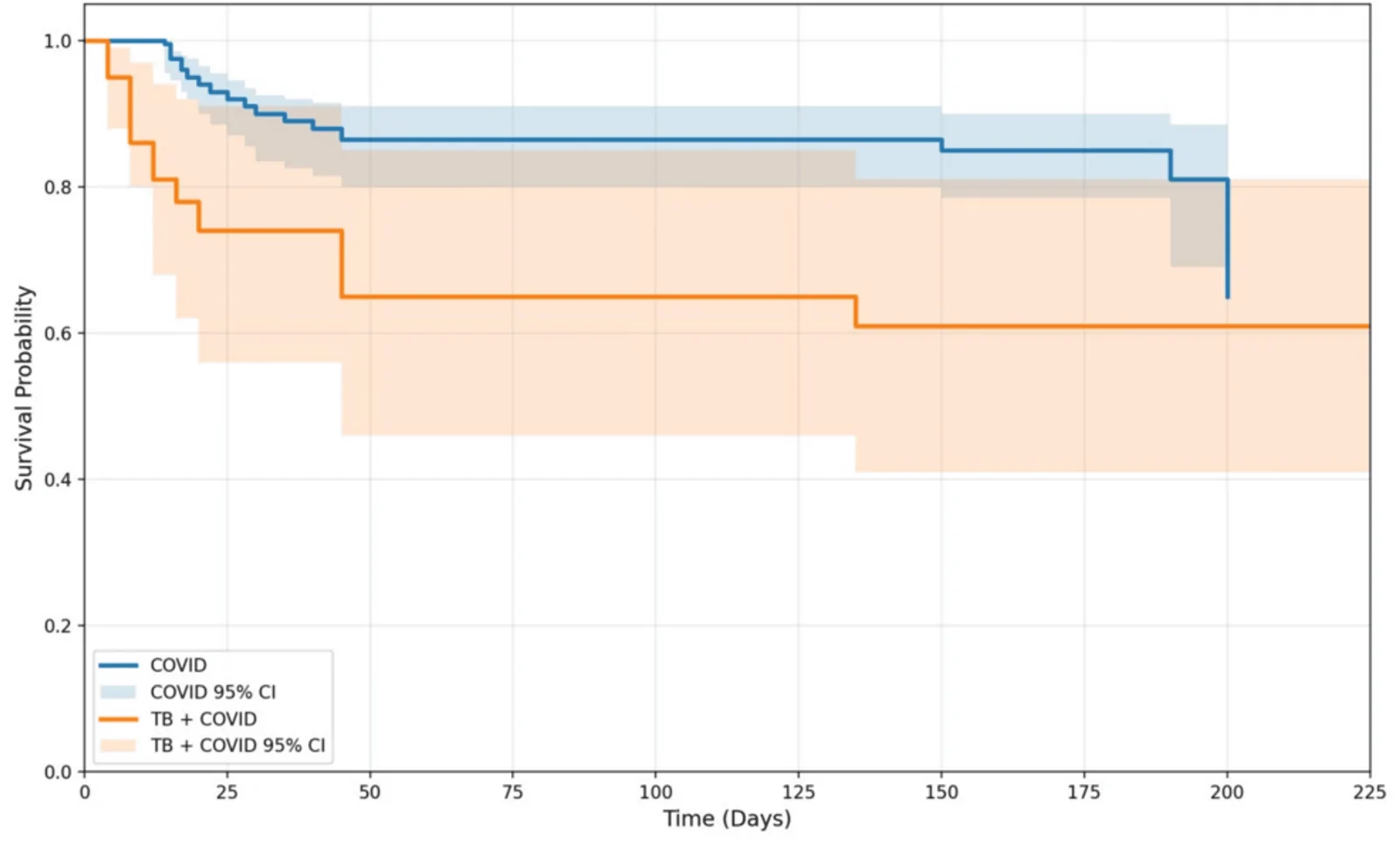
Kaplan-Meier survival comparison between COVID-19 and TB-COVID coinfection Kaplan-Meier survival analysis demonstrated a significant difference in survival between the COVID-19 and TB + COVID-19 groups (log-rank χ² = 6.678, p = 0.0097). The survival curve for the TB + COVID-19 group showed a steeper and earlier decline in survival probability than the COVID-19-only group, indicating greater and earlier mortality among patients with coexisting tuberculosis.

**Figure 4:**
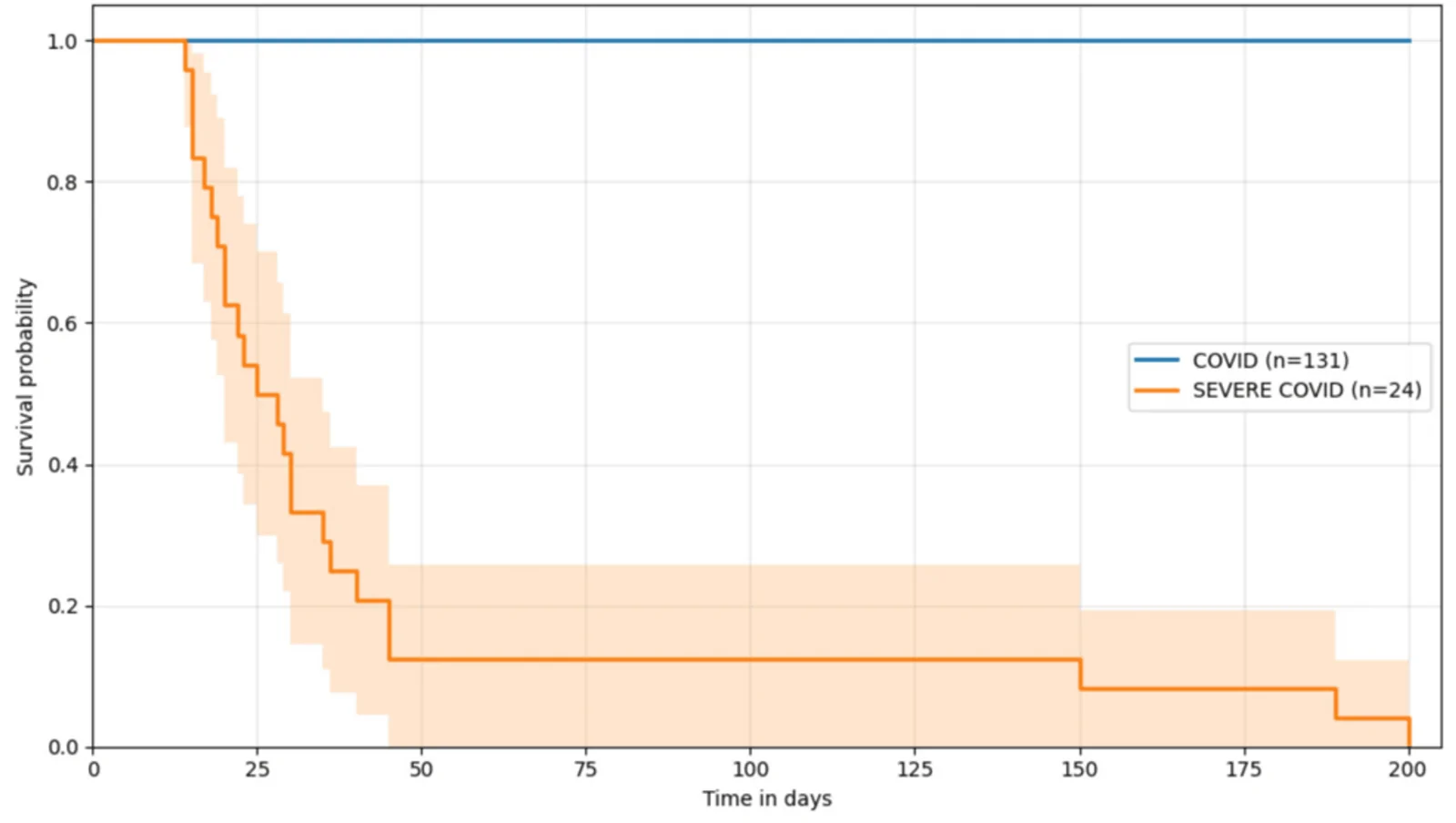
Kaplan-Meier survival curves with 95% CI comparing COVID-19 and severe COVID-19 Kaplan-Meier survival analysis with log-rank testing showed a statistically significant difference in survival between the COVID-19 and severe COVID-19 groups (log-rank χ² = 224.82, df = 1, p < 0.001), indicating poorer survival in the severe COVID-19 group.

Inflammatory biomarkers

No statistically significant difference was detected between patients with COVID-19 alone and those with TB-COVID coinfection in IL-6, ferritin, C-reactive protein, or procalcitonin (all p > 0.05), although median IL-6 was numerically higher in the coinfected group (56.63 vs. 26.30 pg/mL; p = 0.059) (Table 2). Cerebrospinal fluid parameters were available for only one or two coinfected patients and are therefore reported descriptively.

## Discussion

Our study demonstrates that microbiologically confirmed TB-COVID coinfection is associated with higher in-hospital mortality and poorer survival among hospitalized COVID-19 patients with clinical suspicion of TB. The early diagnosis of most TB cases underscores the importance of prompt microbiological evaluation in TB-endemic settings [1-5].

The proportion of microbiologically confirmed TB in our cohort (15.5%) was higher than the 1-10% reported in other studies [4,5,11,13-15]. This difference is expected, because our study included only hospitalized COVID-19 patients with clinical suspicion of TB rather than all hospitalized COVID-19 patients; it also likely reflects India’s high TB burden and the tertiary-care referral setting [3]. This proportion should therefore not be interpreted as the prevalence of TB among all hospitalized COVID-19 patients. Microbiological confirmation using standardized diagnostic methods further strengthens the validity of our findings.

TB may remain unrecognized in COVID-19 patients due to overlapping clinical and radiological features [4,6,7]. In our study, two-thirds of TB cases were diagnosed within seven days of COVID-19 diagnosis, suggesting that many patients had previously undiagnosed TB. However, SARS-CoV-2-induced immune dysregulation and corticosteroid therapy may also contribute to progression or reactivation of latent TB [4,10,11,20]. These findings show the importance of early microbiological evaluation in patients who are hospitalized with clinically suspected TB.

Pulmonary TB was responsible for two-thirds of microbiologically confirmed cases, while one-third had extrapulmonary disease, consistent with reports from tertiary-care centres [7,8]. The substantial proportion of extrapulmonary TB highlights the diverse clinical spectrum of TB and the diagnostic challenges posed by its nonspecific presentation in COVID-19. These study findings emphasize the need to consider extrapulmonary TB in patients who are hospitalized with compatible clinical features.

GeneXpert MTB/RIF demonstrated the highest diagnostic yield, supporting current WHO recommendations for rapid molecular diagnosis of TB [14,21]. Smear microscopy was performed on too few specimens (n = 4) to allow any assessment of its diagnostic performance in this cohort. MPT64 PCR provided additional diagnostic yield, confirming TB in six patients [22]. Overall, the results suggest that molecular testing is needed for early TB diagnosis in hospitalised patients with COVID-19.

In-hospital mortality was approximately twice as high among TB-COVID coinfected patients as among those with COVID-19 alone (37.5% vs. 18.3%; OR 2.68, 95% CI 1.05-6.83), although the wide confidence interval and the borderline result with Fisher’s exact test (p = 0.054) reflect the small number of coinfected patients, so this difference should be interpreted with caution. This result is consistent with previous findings linking active TB to severe COVID-19 and death [5,11,13,23,24]. The increased mortality is believed to be due to the combined effects of pre-existing lung injury and immunological dysregulation associated with both disorders [4,9,25].

Higher mortality with TB-COVID coinfection than with COVID-19 alone has also been documented in India and South Africa [24,26]. Active TB has also been found to be a predictor of severe COVID-19 and death in recent systematic reviews and meta-analysis [5,11,13]. Our study adds microbiologically confirmed data from a high TB-burden setting to the evidence associating TB-COVID coinfection with poor outcomes, although our unadjusted analysis cannot establish a causal effect of TB on mortality.

An important observation was that six of the nine patients who died were diagnosed with TB within one day of death and therefore could not receive adequate anti-tubercular therapy. This finding points to the implications of delayed diagnosis and highlights the importance of early microbiological investigation for TB in patients who are diagnosed with COVID-19 and compatible clinical or radiological features [4,6,23]. In this unadjusted Kaplan-Meier analysis, patients with TB-COVID coinfection had significantly lower survival than those with COVID-19 alone, suggesting a negative prognostic impact of active TB, although few studies have evaluated time-to-event outcomes in microbiologically confirmed TB-COVID cohorts. Our findings support prior publications showing lower survival in coinfected individuals [25-27]. Early diagnosis through rapid molecular testing may facilitate timely initiation of anti-tubercular therapy and potentially improve clinical outcomes.

Patients who had severe COVID-19 were prone to significantly poorer survival than those with non-severe disease, consistent with previous reports identifying severe pulmonary involvement and respiratory failure as major predictors of mortality [20,28]. The coexistence of severe COVID-19 and active TB may further worsen prognosis, highlighting the need for early multidisciplinary management in this high-risk group.

No significant difference in inflammatory biomarkers was detected between patients with COVID-19 alone and those with TB-COVID coinfection (Table 2). However, these comparisons included at most 18 coinfected patients for any parameter, and fewer for some, so the study had limited statistical power to detect modest differences, and the absence of a statistically significant difference should not be interpreted as evidence that the groups are equivalent. Within these constraints, conventional inflammatory markers did not appear to discriminate TB-COVID coinfection and should be interpreted alongside clinical, radiological, and microbiological findings [29,30].

Our findings have important clinical implications. In TB-endemic regions, hospitalized patients with COVID-19 should be evaluated promptly for TB when clinically suspected, with early use of rapid molecular diagnostics to facilitate timely treatment. The poorer survival observed among coinfected patients also supports an integrated approach to the management of both diseases.

The main strength of this study is microbiological confirmation of TB by GeneXpert MTB/RIF, MPT64 PCR, and MGIT 960 culture, whereas many earlier reports relied on clinical diagnosis; both pulmonary and extrapulmonary disease were also included. Limitations include the single-centre design; the small number of coinfected patients, which limited statistical power and precluded multivariable adjustment for confounders such as COVID-19 severity and comorbidities; and the absence of follow-up after discharge. In addition, because TB was diagnosed after the start of follow-up in one-third of coinfected patients, the fixed-group survival analysis is susceptible to immortal-time bias; this bias would be expected to favour the coinfected group, so the observed survival difference is more likely to be underestimated than overstated, but confirmation using time-dependent methods in a larger cohort is warranted. TB testing was performed at the treating clinicians’ request and not every specimen underwent every test, so some TB cases may have been missed and classified as COVID-19 alone; such misclassification would most likely dilute differences between the groups. Laboratory parameters were not available for all patients, which may have introduced selection bias into the biomarker comparisons. Because all comparisons were unadjusted, our findings indicate an association between TB-COVID coinfection and mortality and do not establish a causal effect. Finally, the study population comprised COVID-19 patients with clinical suspicion of TB, so the observed proportion of confirmed TB cannot be extrapolated to all hospitalized COVID-19 patients.

## Conclusions

In conclusion, this prospective study shows that microbiologically confirmed TB was identified in a substantial proportion of hospitalized COVID-19 patients with clinical suspicion of TB in a high TB-burden setting, and that coinfection was associated with higher in-hospital mortality and poorer survival than COVID-19 alone. Most TB cases were identified early through molecular and culture-based diagnostics, yet a considerable proportion of coinfected patients died within a day of TB diagnosis, underscoring how diagnostic delay directly narrows the window for timely anti-tubercular therapy. These findings add to the evidence that active TB is associated with worse outcomes in patients with COVID-19 in endemic populations.

Taken together, these results support a low threshold for microbiological evaluation for TB in hospitalized COVID-19 patients with compatible clinical or radiological features in TB-endemic regions, using rapid molecular diagnostics such as GeneXpert MTB/RIF to enable earlier initiation of treatment. Given the single-centre design and the modest number of coinfected patients, these findings warrant validation in larger, multicentre cohorts with extended post-discharge follow-up. Nonetheless, this study contributes microbiologically confirmed, prospectively collected survival data to a literature that has relied largely on retrospective or clinically diagnosed cohorts, and supports an integrated approach to the clinical management of coexisting COVID-19 and TB in high-burden settings.
